# Ollier Disease: A Case Report and Review of Treatment Options

**DOI:** 10.7759/cureus.43815

**Published:** 2023-08-20

**Authors:** Hunter D Kramer, Michael J Valentine, Nicholas Pettinelli, James Kim, Robert C Kramer

**Affiliations:** 1 College of Medicine, Kansas City University, Kansas City, USA; 2 Hand Surgery, Beaumont Bone & Joint Institute, Beaumont, USA

**Keywords:** bone cancer surgery, lytic bone lesion, pathologic fracture, multiple enchondromatosis, ollier disease, hand enchondromas

## Abstract

Ollier disease is a rare skeletal dysplasia characterized by the formation of multiple enchondromas (enchondromatosis), typically in the long bones of the extremities. These tumors are benign but can become complicated by the development of pathologic fractures, limb deformity, and malignant transformation to chondrosarcoma. Ollier disease has a highly variable presentation and is associated with a range of presenting findings; however, the most common presentation is a pathologic fracture. Surgical options include curettage and grafting of the enchondromas and, when displaced, fracture reduction and fixation. Of note, these fractures will heal without surgery. Regardless, all patients must be routinely monitored with yearly radiographs in order to detect malignant transformation as early as possible.

In this report, we describe the case of an 11-year-old female who presented to her physician with pain and swelling of her right ring and small fingers after playing in a swimming pool with no obvious mechanism of trauma. A routine, plain radiographic evaluation of her hand revealed the presence of multiple enchondromatosis. We hope to use this case to highlight the surgical management options for young patients with Ollier disease and discuss circumstances in which surgical management may not be indicated.

## Introduction

Ollier disease is a rare congenital skeletal disorder that typically affects children under the age of 10. It is characterized by the development of multiple enchondromas in the medullary cavity of the long bones of the hands, the tibia, and, less commonly, in the pelvis. Still, they can occur elsewhere in the appendicular skeleton [1-3]. Enchondromas are benign tumors but can transform into malignant chondrosarcomas in roughly 40% of patients.

The distribution of lesions in enchondromatosis is widely variable but commonly presents predominantly on one side of the body [1]. Of interest, one multicenter study reported an atypical subset of Ollier disease in which enchondroma distribution occurred along a single nerve or single ray [4]. The distribution of lesions may be useful in determining the prognosis. Another international multicenter study reported the progression of Ollier disease into chondrosarcoma, elucidating the likelihood of lesion recurrence and the need for preventative management. Patients with more widespread enchondromatosis that involves the extremities and pelvis were approximately three times more likely to experience malignant transformation compared to patients with predominantly eccentric lesions [5]. 

Due to this variability in location, this disease may require surgical intervention. However, surgical management of metaphyseal enchondromatosis introduces a higher risk of physeal injury, resulting in premature physeal arrest. In these cases, with a stable or nondisplaced fracture pattern, non-surgical options are often explored so as not to interfere with normal skeletal growth.

Male and female children under the age of 10 are more likely to be affected, albeit with a prevalence rate of one in 100,000 [1-2]. While the underlying cause of Ollier disease is unknown, current literature postulates that the etiology of Ollier disease is related to a gain-of-function mutation in the isocitrate dehydrogenases one and two (IDH1 and IDH2). This mutation is sporadic and not heritable [6].

A differential diagnosis may include Maffucci syndrome and Multiple Hereditary Exostoses (MHE). Maffucci’s syndrome is another cause of enchondromatosis but can be differentiated from Ollier disease by the presence of hemangiomas. Multiple Hereditary Exostosis is an autosomal dominant condition characterized by the development of numerous osteochondromas. In contrast to the enchondromas that are characteristic of Ollier disease, osteochondromas tend to grow at the bone surface in an exophytic pattern [5,7].

## Case presentation

**Figure 1:**
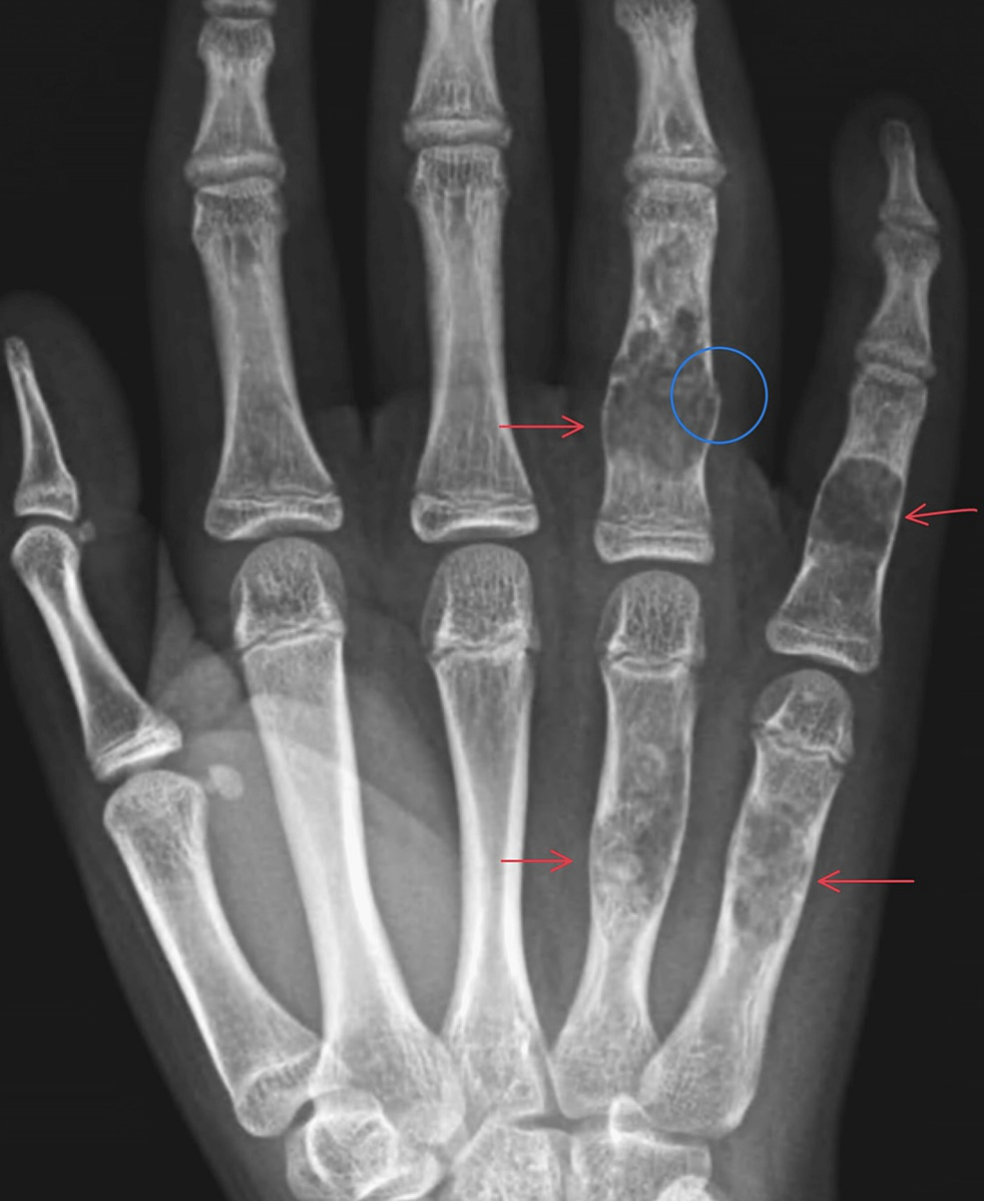
PA view of the right hand Enchondromas (red arrows) are visible in the fourth and fifth proximal phalanges and in the distal to mid-diaphysis of the right fourth and fifth metacarpals. The lesions are circumscribed, expansile, lytic bone lesions with a narrow zone of transition. There is an associated nondisplaced fracture (blue circle) through the dorsal cortex of the fourth proximal phalanx. There is no associated dislocation or osseous erosion, and the joint spaces are preserved.

**Figure 2:**
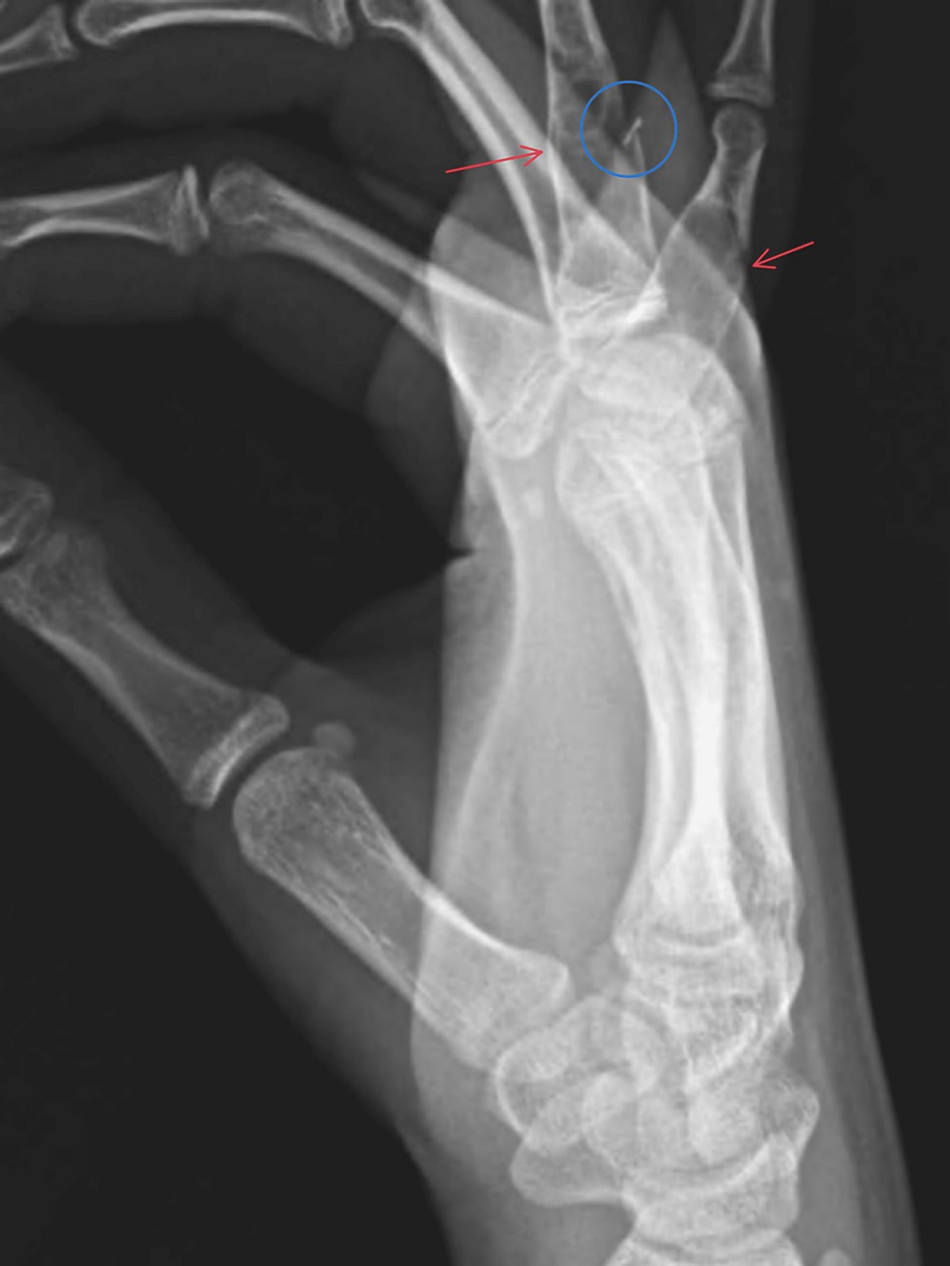
Lateral view of the right hand The lateral view provides a clearer view of the pathologic fracture of the fourth proximal phalanx (blue circle) however, the lesions in the metacarpals are less visible.

A healthy, active 11-year-old female presented to an outpatient clinic with pain and swelling of the right ring finger that began five days prior to the initial evaluation. Pain onset occurred while playing with her friends in a swimming pool. Physical examination demonstrated swelling and focal tenderness to palpation at the base of the right ring finger with faint dorsal ecchymosis and no obvious deformity. There was no observed rotational malalignment. The active range of motion was limited by pain. Radiographs of the finger revealed a non-displaced fracture of the fourth proximal phalanx, secondary to a benign-appearing mid-diaphyseal enchondroma. Additionally, radiographs revealed mid-diaphyseal enchondromatosis in the fifth proximal phalanx and the fourth and fifth metacarpals (Figures 1, 2).

The fracture, being nondisplaced, did not require urgent surgical management and was predicted to heal on its own. Additional radiographic surveys demonstrated no additional sites of pathology. The fracture was immobilized and set for healing. The patient will be followed with plans for eventual corticoplasty and grafting at developmental signs of skeletal maturity.

## Discussion

Physical examination and radiographic findings are paramount in the evaluation and diagnosis of Ollier disease. Physical examination findings are often nonspecific but can reveal the presence of painful nodules, deformity, swelling, and a limb length discrepancy. In addition to a thorough musculoskeletal physical exam, radiographs are necessary to elucidate the presence of multiple enchondromas, which appear as lytic bone lesions in the medullary cavity. Symptomatic sequelae are most commonly the result of pathological progression.

Current literature reports symptoms that may include bone pain, palpable nodules of the extremities, unequal limb lengths, and pathological fractures [1,8]. Therefore, the presentation of this disorder can vary broadly. While this is a rare disease, clinicians should include Ollier disease as part of their differential diagnosis in children who present with seemingly out-of-place fractures from non-traumatic etiology, such as playing in a swimming pool. 

While not required, a biopsy may be obtained for a definitive diagnosis. In cases where a biopsy is obtained, the microscopic features of the lesions will be consistent with those of mature hyaline cartilage. Enchondromas comprise hypocellular, mature hyaline cartilage with a lobular pattern and small chondrocytes [9]. However, in this case, the clinical manifestations and the radiographs were sufficient to make the diagnosis.

Unfortunately, the literature reports an absence of effective drug therapy for Ollier disease, leaving surgical intervention as the primary option for the management of complications [1]. Consequently, due to the variety of manifestations of Ollier disease, many routes must be considered when developing a treatment plan. The primary surgical techniques utilized in this disease are corticoplasty, bone grafting, internal fixation, intramedullary nailing, external fixation, and, rarely, amputation.

Surgical intervention is primarily considered in cases of pathological fractures and tumor-induced limb deformities. The selection of the surgical technique depends on fracture stability or displacement. Corticoplasty involves the creation of a stable cortical window through which tumors can be curettaged and grafted. This procedure has demonstrated good results with a low rate of complications; however, premature closure of the growth plates and enchondroma recurrence have occurred [10-11]. 

Patients with limb deformities from Ollier disease have been successfully treated [12]. Surgical intervention includes bone lengthening, with reports of success from utilizing the Ilizarov external fixation technique [13-14]. In addition to the Ilizarov technique, intramedullary nailing has also been successfully utilized to treat the deformities and limb length discrepancies seen in Ollier disease. In contrast to the Ilizarov method, this technique relies on internal fixation. The elastic, stable intramedullary nail system and the flexible intramedullary nail technique are both techniques that have been successfully utilized. Of note, the literature reports that a combined technique that employs both the Ilizarov technique and flexible intramedullary nailing has been used to improve results and reduce complications [11]. In patients with a significant degree of deformity or extensive tumor expansion, amputation must also be considered as a means to maximize survival.

In a long-term follow-up study of multiple enchondromas in the hands of 12 children, Kadar et al. conclude that pediatric populations with Ollier disease of the hand have low malignancy rates with a good prognosis [15]. They reported that the most common complication was lesion recurrence at a rate of 33% (four out of 12) and specifically 18% (seven out of 38) of the lesions, with the possibility of phalangeal growth arrest. 

In this case, an 11-year-old female presented with finger pain after playing in the swimming pool, having sustained no apparent trauma. This story prompted further work. Radiographic imaging revealed a nondisplaced pathologic fracture and the incidental discovery of diaphyseal enchondromatosis involving the ring and small fingers of her dominant hand. There was no evidence of hemangiomas, confirming Ollier’s diagnosis.

Considering her fracture was stable, surgery was not immediately indicated. The fracture was immobilized, with follow-up until healing. Additional work-up confirmed a disease limited to her right hand. She will be followed closely with planned curettage and grafting at, or near, skeletal maturity in hopes of preventing malignant transformation.

## Conclusions

Ollier disease is extremely rare. Its presentation is highly variable and can range from an incidental finding to pathological fractures to limb length discrepancies. This variability can make the diagnosis hard to catch. Additionally, clinical management is complicated and must be determined based on multiple factors. Correction of deformity and prevention of malignancy are the primary goals of surgery in this disease. Surgical treatment involves curettage, bone grafting and, in the most severe cases, may require limb-lengthening procedures or amputation. Conservative management must also be considered. 
